# Exosome-Derived circ0009910 Promotes Pituitary Adenoma Cell Proliferation, Invasion, Migration, and EMT through the miR-106b-5p/STAT3 Axis

**DOI:** 10.1055/a-2599-4212

**Published:** 2025-09-18

**Authors:** Zexu X. Yang, Wenge G. Zhang, Leiguo G. Wei, Yufei F. Qu, Jiazi Z. Yin, Qi Liu

**Affiliations:** 1 Department of Neurosurgery, The First Affiliated Hospital of Shihezi University, Shihezi, Xinjiang, People's Republic of China

**Keywords:** pituitary adenoma, exosomes, circ0009910, invasion

## Abstract

**Objectives:**

This study aimed to explore whether exosome-derived circ0009910 can transcellularly regulate the growth of pituitary adenoma (PA) cells and to further explore the possible mechanisms of its action.

**Methods:**

Transmission electron microscopy and nanoparticle size analysis were used to observe the morphology and size of the exosomes. Real-time quantitative polymerase chain reaction (qRT-PCR) was used to determine the expression of circ_0009910, miR-106b-5p, and signal transducer and activator of transcription3 (STAT3). Western blotting was used to assess the expression of exosomal marker proteins, p-STAT3, E-cadherin, N-cadherin, and vimentin. Cell Counting Kit-8 (CCK-8) and 5-Ethynyl-2-deoxyuridine (EdU) assays were used to determine the proliferative capacity of the cells. Transwell assays were performed to assess the migratory and invasive capacity of the cells. Enzyme-linked immunosorbent assays (ELISAs) were used to determine the expression level of growth hormone (GH). A nude mouse xenograft model was established to observe the effects of exosome-derived circ0009910 on transplanted tumors in nude mice.

**Results:**

circ0009910 can be transferred to other cells via exosomes. Knocking down the expression of circ0009910 can inhibit the proliferation, invasion, and migration of PA cells, reduce GH expression, and regulate the expression of epithelial–mesenchymal transition (EMT)-associated proteins. miR-106b-5p is a molecular sponge of circ0009910 and can partially reverse the procarcinogenic effect of circ0009910 in PA. *STAT3* is a target gene of miR-106b-5p. In addition, circ0009910 knockdown inhibited tumor growth in vivo.

**Conclusion:**

Exosome-derived circ0009910 promotes PA progression and regulates EMT through the miR-106b-5p/STAT3 axis.

## Introduction

Pituitary adenomas (PAs) are common benign tumors, accounting for approximately 15% of intracranial tumors. 1 Some PAs show characteristics of benign tumors, grow slowly, are confined to the sella, and display good regression after surgery or drug treatment. However, 30 to 40% of PAs invade important structures, such as the cavernous sinus, pterygoid ridge, and carotid arteries and show a higher rate of proliferation. This type of PA is known as an invasive PA (IPA). 2 IPAs are difficult to operate on and tend to show recurrence after surgery, and the survival rate of patients after surgery is significantly reduced. 3 Therefore, exploration of the possible pathogenesis of IPAs is needed to improve the diagnosis and treatment of this condition.

Exosomes are extracellular vesicles that widely exist in eukaryotes and prokaryotes, with a diameter between 30 and 150 nm, and are rich in nucleic acids, proteins, lipids, and other biologically active substances. 4 The unique double-layer plasma membrane structure of exosomes can better protect their contents from degradation. As good carriers, exosomes play an important role in mediating intercellular communication, 5 6 immunosuppression, 7 8 cardiovascular and cerebrovascular diseases, 9 and tumor development. 10 11 Recent studies have shown that tumor cells secrete more exosomes than normal cells, and these exosomes may alter tumor properties by delivering the genetic material they contain. 12 However, the role played by exosomes in the invasion progression of PA remains unclear.

Our previous study found that the expression level of serum circ0009910 was increased in patients with IPA, and knockdown of circ0009910 could inhibit the growth of PA cells. 13 During the experiment, we unexpectedly found that circ0009910 knockdown in PA cell supernatant inhibited the growth of normal PA cells. Although there are some recent reports on circ0009910, 14 15 16 the role of exosomal circ0009910 in PA has not been reported. Therefore, we further investigated whether circ0009910 is contained in exosomes and shuttles in the tumor microenvironment to exert its growth-promoting effect on PA.

In this study, we demonstrated that circ0009910 is present in exosomes and can be transferred to other cells with exosomes. Downregulation of circ0009910 inhibited PA cell proliferation, invasion, and migration and led to increased miR-106b-5p expression levels. Overexpression of miR-106b-5p inhibited the adverse biological behavior of PA cells by targeting signal transducer and activator of transcription3 (STAT3). The above findings suggest that circ0009910 may be a novel molecular marker for the diagnosis of IPA.

## Materials and Methods

### Patient Information and Blood Specimen Collection

This study was approved by the Ethics Committee of the First Affiliated Hospital of Shihezi University (approval number KJX2022-062-01), and all experiments in the study complied with the principles of the Declaration of Helsinki. We enrolled patients with PAs who were admitted to the Department of Neurosurgery at the First Affiliated Hospital of Shihezi University from October 2020 to October 2023, totaling 65 cases. All patients participating in the study signed an informed consent form. The diagnosis of PA was made by specialists from the Department of Neurosurgery, Department of Imaging, and Department of Pathology of the First Affiliated Hospital of Shihezi University, using magnetic resonance imaging of the sellar region, serum hormone levels, and pathological findings.

### Growth Hormone and Prolactin Expression Levels Detection Assays

Venous blood was collected from patients on an empty stomach and in a quiet state. The fully automated particle chemiluminescence immunoassay system (Genstar, Beijing, China) was used to detect growth hormone (GH) and prolactin (PRL) levels.

### Cell Culture and Transfection

Rat PA cell lines (GH3, MMQ) were purchased from Wuhan Boster Co. (Hubei, China) and cultured at 37 °C, 90% humidity, and 5% CO _2_ . These cells were cultured in F12K culture medium containing 2.5% fetal bovine serum (FBS), 15% horse serum, and 1% penicillin/streptomycin, and the culture medium was changed every 48 hours. circ_0009910 knockdown lentivirus was purchased from GenePharma (GenePharma Corporation, Shanghai, China), transfection was performed according to the manufacturer's instructions with the assistance of polybrene, and PA cells were screened with 3 μg/mL puromycin (Sigma–Aldrich, Zhangzhou, Fujian, China) for 2 weeks after completion of transfection. Small interfering RNA (miR-106b-5p mimic, miR-106b-5p inhibitor, NC) was purchased from GenePharma (GenePharma Corporation, Shanghai, China) and used with Lipofectamine 3000 (Thermo Fisher Scientific, Inc., MA) for transfection.

### Xenograft Animal Models

All animal work was approved by the Laboratory Animal Ethics Committee of the First Affiliated Hospital of Shihezi University (approval number A2022-202-01) and was conducted in accordance with the Guidelines for the Care and Use of Laboratory Animals in the National Institutes of Health. Sterile female nude mice, 4 to 6 weeks of age, were purchased from Henan Skebes Biotechnology Co. (Henan, China) and were assessed by Suzhou Sisan Biotechnology Co., Ltd. (Laboratory Animal License No. SCXK [Yu] 2020-0005]. The nude mice were kept under specific pathogen-free conditions. Normal GH3 cells (400 × 10 ^4^ ), circ_0009910 knockdown GH3 cells (400 × 10 ^4^ ), or empty vector lentiviral GH3 cells (400 × 10 ^4^ ) were mixed in phosphate buffer solution (PBS) and injected subcutaneously into nude mice. Xenograft volume was assessed by two vertical diameter caliper measurements and calculated using the formula volume (V) = a × b ^2^ /2, where “a” represents length and “b” represents width. The weight of the nude mice as well as the size of the tumors was measured every other day. All nude mice were sacrificed after 14 days, and tumor samples were collected and photographed.

For the exosome treatment model, conditioned cultures of circ_0009910 knockdown GH3 cells were collected, exosomes were isolated by ultracentrifugation and then lysed using PBS, and xenograft tumors were established at 400 × 10 ^4^ GH3 cells per nude mouse. Nude mice were randomly divided into two groups: the control and the circ0009910 knockdown. Exosomes (100 μg of total protein in a volume of 100 μL) were injected twice a week via the tail vein, and tumor volume was calculated as described above.

### Exosome Extraction

Exosomes were collected by continuous ultracentrifugation. GH3 cells were cultured in exosome-free complete medium for 48 hours. The culture supernatant was collected, and debris and apoptotic vesicles were first removed from the supernatant by centrifugation at 2,000 × g (20 minutes) and 10,000 × g (30 minutes). The supernatant was collected and ultracentrifuged at 110,000 × g for 70 minutes, and the supernatant was discarded. Exosomes were washed with sterilized PBS and purified by centrifugation at 110,000 × g for 70 minutes. After the exosomes were resuspended in PBS and filtered through a 0.22-mm filter (Millex-HV, Sigma–Aldrich, Fujian, China), the samples were stored at −80 °C.

### Transmission Electron Microscopy

The morphology of exosomes was observed under a transmission electron microscope with negative staining (Hitachi, Tokyo, Japan). A drop of exosome suspension (∼10 μL) was incubated on a copper grid for 1 minute, dried at 65 °C, and observed using a transmission electron microscope equipped with an HT7700 with an accelerating voltage of 80 kV. Images were acquired using a charge-coupled device (Gatan, Pleasanton, CA).

### Nanoparticle Tracer Analysis

The purified exosomes were resuspended in 100 μL of 0.22 μm-filtered PBS. A nanoparticle tracking analyzer (Particle Metrix, ZetaView) was used to determine the particle size and concentration of exosomes, which were statistically analyzed using Nanoparticle Tracer Analysis (NTA) 3.0 software. Each sample was analyzed independently three times.

### Exosome Tracking

Cells were cultured using six-well plates, coverslips were placed into the plates to complete the cell crawls, exosomes were extracted from cell supernatants using ultracentrifugation, protein quantification was performed by the bicinchoninic acid (BCA) method, exosomes were labeled using PHK26 (Maokang Biotechnology, Shanghai, China) and then ultracentrifuged again, and the precipitate was resuspended in PBS and added to the six-well plates to be cultured for 24 hours. The nuclei of cells were stained with an antifluorescence quencher containing 4′ 6-diamidino-2-phenylindole (Solarbio, China) and observed under a fluorescence microscope.

### Quantitative Reverse Transcription Polymerase Chain Reaction

The expression of circ0009910, miR-106b-5p, and STAT3 was determined in each group by quantitative reverse transcription polymerase chain reaction (qRT-PCR). Total RNA was extracted using the E.Z.N.A Total RNA Kit I (Omega Bio-Tek, Norcross, Georgia) as directed by the manual. cDNA was reverse transcribed using the RevertAid First Strand cDNA Synthesis Kit Reverse-Transcription Kit (Thermo Scientific, Waltham, Massachusetts, USA), and cDNA was extracted using the SYBR Green PCR Kit (SYBR® Green Real-time PCR Master Mix, QIAGEN, Dusseldorf, Germany) for quantification. qRT-PCR primers were provided by GenePharma (GenePharma Corporation, Shanghai, China).

### Cell Counting Kit-8 Assay

The proliferative capacity of GH3 cells was determined using the Cell Counting Kit-8 (CCK-8) assay. After different treatments, cells were inoculated in 96-well plates at a density of 5,000 cells/well, and after 24, 48, and 72 hours of incubation, 10 μL of CCK-8 solution (APE × BIO) was added to each well. The plates were incubated at 37 °C for 3 hours, protected from light, and the absorbance was measured at 450 nm using an enzyme-linked immunoassay analyzer (Thermo Fisher, Beijing, China).

### 5-Ethynyl-2-Deoxyuridine Assay

5-Ethynyl-2-deoxyuridine (EdU) Imaging Kits (Cy3; APE × BIO) were used to determine the effect of EdU on GH3 cell proliferation. GH3 cells with different interventions were inoculated in six-well plates and cultured to the wall, and EdU (10 μM) working solution was added and incubated at 37 °C for 2 hours. Fixation, permeabilization, click reaction, and nuclear staining of cells were performed sequentially according to the instruction guide. Finally, EdU-positive cells were observed and analyzed by fluorescence microscopy (ZEISS, Oberkochen, Baden-Württemberg, Germany).

### Colony Formation Assay

GH3 cells after sh-circ0009910 GH3 group and negative control (NC) group supernatant intervention were collected and inoculated in six-well plates at 500 cells per well and incubated at 37 °C for 14 days. After the medium was removed, the cells were washed twice with PBS and fixed with 4% paraformaldehyde for 30 minutes, and the number of colonies was determined after 0.1% crystal violet staining.

### Cell Migration and Invasion Assays

In cell migration assays, 10 ^5^ cells were added to 0.3 μL of F12K serum-free culture medium to generate a cell suspension, which was added to the upper chamber and incubated for 24 hours. Cell invasion assays were performed with Transwell chambers coated with Matrigel (Corning, New York, USA), and 1.5 × 10 ^5^ cells were added to 0.3 μL of F12K serum-free culture medium to generate a suspension. The cell suspension was added to the upper chamber and incubated for 24 hours. Cells were fixed with formaldehyde for 30 minutes and stained with crystal violet, and the number of cells crossing the filter membrane was counted under the microscope after washing with PBS.

### Immunohistochemistry

Tissues were fixed in 10% formalin, embedded, and cut into 4-μm-thick sections for EnVision two-step immunohistochemical staining. Sections were deparaffinized and hydrated with ethanol. Endogenous peroxidase was removed by microwave antigen repair and hydrogen peroxide. After washes with PBS, primary antibodies (rabbit polyclonal GH antibody, 1:200; rabbit monoclonal PRL antibody, 1:50; rabbit polyclonal Pit-11:200 antibody; mouse monoclonal P53 antibody, 1:200; mouse monoclonal ER antibody, 1:100; rabbit monoclonal GATA-2 antibody, 1:20; Boster) were added, and the sections were incubated overnight at 4 °C, rewarmed at 37 °C for 30 minutes, and then rinsed with PBS. After dropwise addition of secondary antibody, incubation was performed for 30 minutes, the sections were rinsed with PBS, and then, diaminobenzidine color development was performed. After hematoxylin staining and acidic ethanol acidification, the antibody color turned blue, and the samples were cleared with xylene. The samples were dried at 37 °C, sealed with neutral adhesive, and observed under a microscope. The product of the staining intensity and the staining range score was used as the immunohistochemistry (IHC) score. Two pathologists reviewed the sections.

### Bicinchoninic Acid Assay

According to the standard and sample quantity, the BCA working solution was prepared by adding 50 volumes of BCA reagent and 1 volume of Cu reagent (50:1). Ten microliters of BSA standard was diluted with PBS to 100 μL, resulting in a final concentration of 0.5 mg/mL. Then, 0, 2, 4, 6, 8, 12, 16, and 20 μL of the standard substance was added to the protein standard wells of a 96-well plate, and PBS was added to make the volume up to 20 μL equivalent to standard concentrations of 0, 0.05, 0.1, 0.15, 0.2, 0.3, 0.4, and 0.5 mg/mL, respectively. The sample was diluted appropriately, and 20 μL was added to the sample well of the 96-well plate. About 200 μL of BCA working solution was added to each well and allowed to stand at 37 °C for 15 to 30 minutes. A562 nm was measured using an enzyme-linked immunosorbent assay (ELISA) reader and the protein concentration was calculated based on the standard curve.

### Western Blot Analysis

All cellular proteins were extracted from radio immunoprecipitation assay lysis buffer (Solarbio, Beijing, China) for gel electrophoresis. Isolated proteins were transferred to polyvinylidene fluoride membranes (microtiter wells, [Solarbio, Beijing, China]) at 200 mA, and then, the cell membranes were blocked with 5% skim milk powder for 1 hour at room temperature, followed by overnight incubation with primary antibody at 4 °C. The next day, the membranes were conjugated with secondary antibody (1:5,000) for 1 hour at room temperature. Finally, each protein band was visualized with an enhanced chemiluminescence kit (Pierce Biotechnology; Thermo Fisher Scientific, Inc., Massachusetts, USA), and the relevant data were quantified with Image Laboratory software.

### Statistical Analysis

Each experiment was repeated three times, and the experimental data are expressed as the mean ± standard deviation (SD). The results were mainly analyzed using SPSS 26.0 statistical software. An unpaired two-tailed *t* -test was used to calculate the significant differences between the control and experimental groups. Other statistical variables between multiple groups were assessed using one-way analysis of variance (ANOVA). *p* -Value <0.05 was considered statistically significant.

## Results

### Serum Exosomal circ0009910 Expression in Pituitary Adenoma Patients

The data of 65 patients with PAs were collected, and the basic data of the enrolled patients are shown in Table 1 .

**Table 1:** Clinical data of 65 patients with pituitary adenomas

Clinical data	Cases ( *n* ,%)
Invasive classification	
IPAs	40 (61.5)
Noninvasive PAs	25 (38.5)
Sex	
Male	27 (41.5)
Female	38 (58.5)
Knosp grading	
I	6 (9.2)
II	19 (29.2)
III	18 (27.7)
IV	22 (33.8)
Ki67	
≥ 3%	28 (43.1)
< 3%	37 (56.9)

Abbreviation: IPA, invasive pituitary adenoma.

**Fig. 1:**
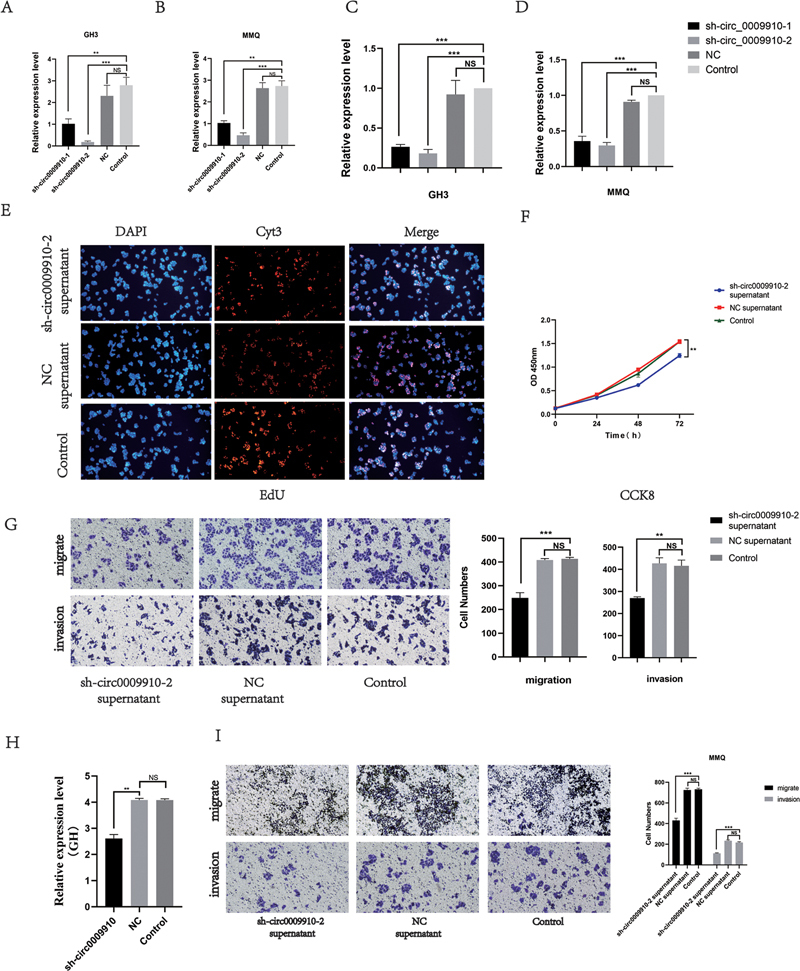
Exosome circ_0009910 inhibits PA cell growth. **(A)** Exosome circ_0009910 is highly expressed in the serum of PA patients. **(B)** Exosome circ_0009910 is expressed at a higher level in the serum of patients with IPAs than those with noninvasive PAs. **(C, D)** Transfection efficiency of sh-circ0009910 lentivirus in GH3 and MMQ cells. **(E, F)** sh-circ0009910–2 cell supernatant inhibits the proliferation of normal GH3 cells in vitro. **(G)** sh-circ0009910–2 cell supernatant inhibits invasion and migration of normal GH3 cells in vitro. **(H)** Growth hormone levels are reduced in sh-circ0009910–2 GH3 cell supernatants. **(I)** sh-circ0009910–2 cell supernatant inhibits invasion and migration of normal MMQ cells in vitro. ** *p* < 0.01, *** *p* < 0.001. CCK-8, Cell Counting Kit-8; EdU, 5-ethynyl-2-deoxyuridine; IPA, invasive pituitary adenoma; NS, no statistical difference; PA, pituitary adenoma.

By qRT-PCR, we found that the expression level of circ0009910 was higher in serum exosomes of patients with PAs than in normal controls ( Fig. 1A ), and in patients with IPAs than in PAs ( Fig. 1B ).

Grouping the aggressiveness according to the Knosp classification, patients' age, gender, smoking history, alcohol consumption history, hypertension history, diabetes history, BMI, Ki67, serum PRL level and GH level were not significantly correlated with the aggressiveness of the tumors ( *p* > 0.05). Theexpression level of exosome circ0009910 was significantly correlated with the invasion of the tumors and the difference was statistically significant ( *p* < 0.001; Table 2 ). The primer sequences used for quantitative RT-PCR detection are shown in Supplementary Table 1 (available in the online version), and the antibody information used for immunohistochemistry and Western blotting is shown in Supplementary Table 2 (available in the online version).

**Table 2:** Relationship between the expression of circ_0009910 and clinical data in patients with pituitary adenoma

Parameter	IPA ( *n* = 40)	Noninvasive PA ( *n* = 25)	*χ*^2^ / *t*	*p* -Value
Sex ( *n* )			0.513	0.47
Male	18	9		
Female	22	16		
Age (years)	54.93 ± 12.29	55.20 ± 12.64	−0.09	0.93
Smoking ( *n* )	12 (0.30)	6 (0.24)	0.28	0.60
Alcohol ( *n* )	4 (0.10)	2 (0.08)	0.07	0.79
Hypertension ( *n* )	18 (0.45)	8 (0.32)	1.08	0.30
Diabetes ( *n* )	8 (0.20)	4 (0.16)	0.16	0.69
BMI (kg/m ^2^ )	25.91 ± 3.80	25.53 ± 4.13	0.38	0.71
Ki-67	(2.00, 3.00)	(2.00, 3.00)	0.13	0.72
PRL (ng/mL)	(48.61, 385.36)	(63.4, 223.14)	0.18	0.68
GH (ng/mL)	(0.23, 0.57)	(0.49, 3.44)	2.64	0.10
Exosomal circ_0009910	10.91 ± 6.37	5.85 ± 1.20	4.88	<0.001

Abbreviation: BMI, body mass index; GH, growth hormone; IPA, invasive pituitary adenoma; PRL, prolactin.

*n* is the number of cases. If *p* < 0.05, the difference was statistically significant.

The patients were divided into high and low expression groups according to the optimal cutoff value of exosomal circ0009910 expression level, and the correlation between exosomal circ0009910 expression level and the clinicopathological data of PAs patients are analyzed in Table 3 . The exosomal circ0009910 expression level was significantly correlated with invasiveness ( *χ* ^2^ = 25.656, *p* < 0.001), with no significant correlation with gender, age, and Ki67 level ( *p* > 0.05).

**Table 3:** Correlation of serum exosome circ_0009910 expression with clinicopathologic data in patients with pituitary adenomas

Relevant factor	Exosome circ_0009910		*χ* ^2^	*p* -Value
	High expression	Low expression		
Age (years)			0.195	0.659
< 55	12 (44.4)	15 (55.5)		
≥ 55	19 (0.5)	19 (0.5)		
Sex				
Male	14 (51.9)	13 (48.1)	0.320	0.571
Female	17 (44.7)	21 (55.3)		
Invasive				
IPAs	29 (72.5)	11 (27.5)	25.656	<0.001
Noninvasive PAs	2 (8.0)	23 (92.0)		
Ki67			2.477	0.116
< 3%	16 (59.3)	11 (40.7)		
≥ 3%	15 (39.5)	23 (60.5)		

Abbreviation: IPA, invasive pituitary adenoma.

### circ0009910 Knockdown Cell Medium Inhibits the In Vitro Proliferation, Migration, and Invasion of Pituitary Adenoma Cells

The expression of circ0009910 was knocked down in GH3 and MMQ cells using two short hairpin RNAs (shRNAs). The qRT-PCR results showed a strong inhibitory effect of sh_ circ0009910–2 ( *p* < 0.05; Fig. 1C , D ).

circ0009910 knockdown GH3 cells were cultured, supernatant samples were collected, and normal GH3 cells were treated with the supernatant. The CCK-8 and EdU results showed that the sh_circ0009910–2 GH3 cell supernatant had a significant inhibitory effect on the proliferation of normal GH3 cells compared with the control supernatant ( Fig. 1E , F ). The sh_circ0009910–2 GH3 cell supernatant also effectively inhibited the migration, invasion of normal GH3 cells ( Fig. 1G ). The expression level of GH was decreased in the sh_circ0009910–2 GH3 cell supernatant compared with the GH3 cell supernatant ( Fig. 1H ). Similarly, after treatment with circ0009910–2 MMQ cell supernatant, the migration and invasion of MMQ cells were weakened compared with those before treatment ( Fig. 1I ).

The results of the above experiments suggest that circ0009910 may be secreted out of the cell and shuttled in body fluids to act on other cells.

### Exosomes Serve as the Medium for circ0009910 to Exert Intercellular Communication

**Fig. 2:**
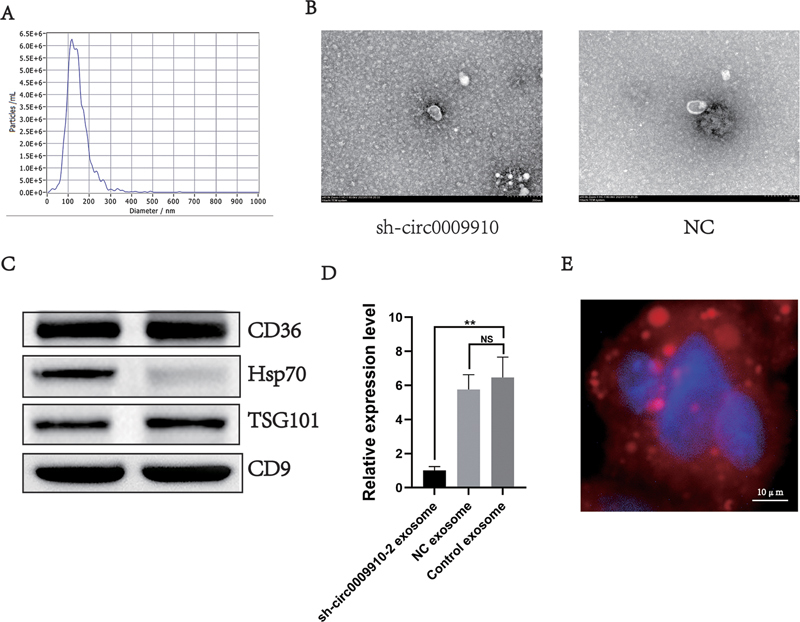
Identification of exosomes from cell supernatants. **(A)** NTA detection of the diameter of exosomes extracted from PA cell supernatants. **(B)** Transmission electron microscopy to observe the morphology of exosomes from the PA cell supernatant extracts. **(C)** WB detection of exosome marker protein expression. **(D)** qRT-PCR to detect the expression of circ_0009910 in each group of exosomes. **(E)** Absorption of normal exosomes by GH3 cells was detected by the exosome tracing method, PKH26-labeled exosomes(red) were added to the cell medium for 24 hours and observed under a fluorescence microscope (scale bar, 10 μm). ** *p* < 0.01, *** *p* < 0.001. NS, no statistical difference; NTA, Nanoparticle Tracer Analysis; PA, pituitary adenoma; qRT-PCR, quantitative reverse transcription polymerase chain reaction; WB, Western blot.

Exosomes are rich in a variety of genetic material, and their role as carriers mediating intercellular communication has been widely reported. Based on the results of previous studies, we hypothesized that circ0009910 may be embedded in exosomes to act on other cells. The supernatants of each group of cell-conditioned cultures were collected, and the exosomes were extracted and analyzed using ultracentrifugation, NTA, and transmission electron microscopy (TEM) combined with WB results, which indicated the presence of exosomes with a typical lipid bilayer membrane structure in the conditioned cultures of all three groups of cells ( Fig. 2A – C ). The expression of circ0009910 in the exosomes of each group was measured by qRT-PCR. The circ0009910 expression level in exosomes from the normal GH3 culture solution was significantly higher than that in the sh-circ0009910–2 GH3 cell group ( Fig. 2D ).

Subsequently, the exosomes secreted by the sh_circ0009910–2 GH3 cells were labeled using lipophilic dyes, and these exosomes were added to the normal GH3 cell culture solution for culture. Exosomes labeled with PKH26 (red) were observed in the cytoplasm of normal GH3 cells ( Fig. 2E ). Therefore, we hypothesized that the exosomes secreted by sh_circ0009910–2 GH3 cells can freely shuttle in the tumor microenvironment and enter nearby GH3 cells to mediate their effects.

These results tentatively suggest that circ0009910 can be secreted out of the cell with exosomes and into other cells to fulfill its function.

### Exosome-Derived circ0009910 Promotes Pituitary Adenoma Growth In Vivo

**Fig. 3:**
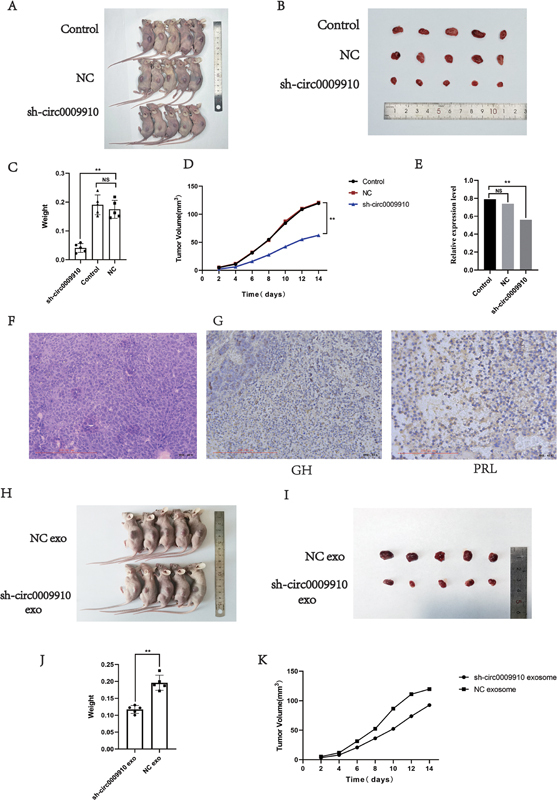
sh-circ0009910 inhibits the growth of pituitary adenomas in vivo. **(A, B)** Each group of nude mice was injected subcutaneously with 4 × 10 ^6^ GH3 cells, cultured for 2 weeks, and then euthanized. Tumors were excised and photographed. **(C)** The tumor volume in the sh-circ0009910 group was smaller than that in the normal GH3 cell group. **(D)** The tumor growth rate of sh-circ0009910 group was smaller than that of the normal GH3 cell group. **(E)** Expression of circ_0009910 in the tumors of each group. **(F)** HE staining of tumor tissues. **(G)** Immunohistochemical detection of GH+ and PRL+ in tumor tissues (scale bar, 100 mm). **(H, I)** Two groups of nude mice were injected with the NC group or sh-circ0009910 group exosomes in the tail vein twice a week, and the tumors were excised and photographed. **(J)** The tumor volume in the sh-circ_0009910 exosome group was smaller than that in the NC exosome group. **(K)** The tumor growth rate in the sh-circ0009910 exosome group was smaller than that in the NC exosome group. ** *p* < 0.01. GH, growth hormone; HE, hematoxylin eosin; NC, NS, no statistical difference; PRL, prolactin.

Nude mice were randomly divided into three groups that received ipsilateral subcutaneous injection of 4 × 10 ^6^ normal GH3 cells, sh_circ0009910–2 GH3 cells, and GH3 cells of the NC group, followed by normal feeding and recording of tumor growth. All nude mice were sacrificed and photographed after 2 weeks ( Fig. 3A , B ), and the tumor size was determined. The results showed that circ0009910 knockdown had a significant inhibitory effect on both the tumor growth rate and tumor weight ( Fig. 3C , D ). The expression level of circ0009910 in the tumors of each group was further analyzed, and as expected, the expression level of circ0009910 was reduced in the tumors of the group inoculated with sh_circ0009910–2 GH3 cells ( Fig. 3E ).

HE staining showed loss of normal morphology of the tissue and small nuclei were visible, and the IHC results were GH+ and PRL + , which were consistent with the type of hormone secreted by GH3 cells, and the tumor could be judged as PA ( Fig. 3F , G ).

For further analysis of the effect of exosomes secreted by the circ0009910 knockdown GH3 cells on the growth of PA in vivo, nude mice were randomly divided into two groups. Exosomes of the control group and exosomes of the sh_circ0009910–2 GH3 cell group were injected into the tail vein (100 μg/100 μL) after tumor development for 1 week, and were injected twice per week. The nude mice were treated with the exosomes of the control group and exosomes of the sh_circ0009910–2 GH3 cell group after 2 weeks of injection and were euthanized and photographed ( Fig. 3H , I ). The experimental results showed that the tumor weight of the nude mice injected with circ0009910 knockdown GH3 cell-secreting exosomes was significantly reduced compared with that of the nude mice injected with control GH3 cell-secreting exosomes, and the tumor growth became slower, and the GH expression level was reduced ( Fig. 3J , K ).

The above in vivo results demonstrate once again that circ0009910 can be contained in exosomes to exert its tumor-promoting effects.

### miR-106b-5p is a Molecular Sponge of circ0009910 that Functions through STAT3

Our group has shown that STAT3 is a molecule closely related to the invasion of PA, 17 and the regulation of GH levels by STAT3 in patients with PA has also been reported in several papers. 18 19 20 In the present study, we found that circ0009910 also plays an important role in regulating the invasion of PA and GH levels, and the link between these two molecules is unclear.

**Fig. 4:**
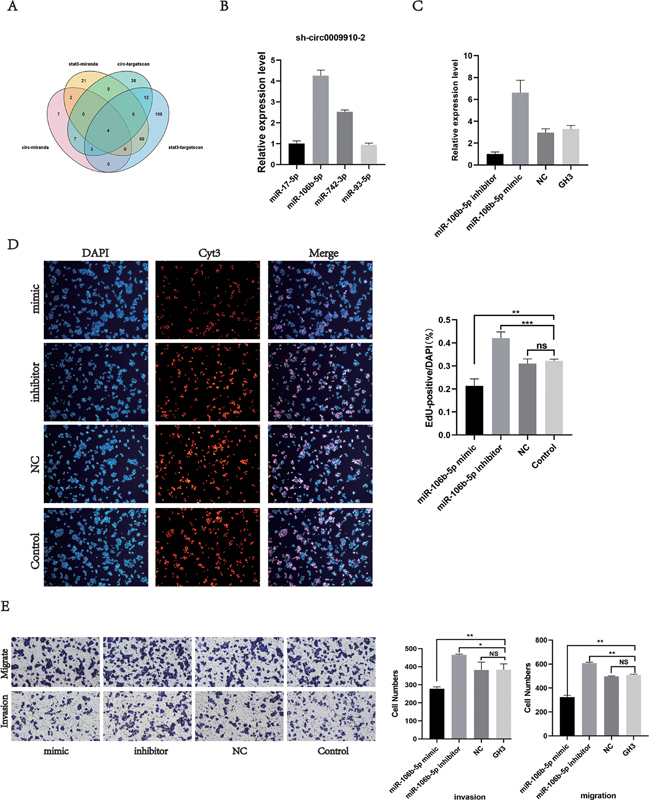
miR-106b-5p is the molecular sponge of circ_0009910. **(A)** TargetScan and miRanda databases were analyzed for possible miRNAs between circ0009910 and STAT3. **(B)** qRT-PCR validation of miR-17-5p, miR-106b-5p, miR-742-3p, and miR-93-5p expression in sh-circ0009910 GH3 cells. **(C)** Transfection efficiency of miR-106b-5p. **(D)** miR-106b-5p mimic inhibits GH3 cell proliferation in vitro. **(E)** miR-106b-5p mimic inhibits GH3 cell invasion and migration in vitro. * *p* < 0.05, ** *p* < 0.01, *** *p* < 0.001. NS, no statistically different; qRT-PCR, quantitative reverse transcription polymerase chain reaction.

Two online databases (TargetScan and miRanda) were utilized to predict the miRNAs of circ0009910 binding to STAT3, and a total of four overlapping genes were found in the databases: miR-17-5p, miR-106b-5p, miR-742-3p, and miR-93-5p ( Fig. 4A ). After analyzing the expression of the above four genes in sh_circ0009910–2 GH3 cells, we found that miR-106b-5p was expressed at a higher level ( Fig. 4B ).

These results suggest that miR-106b-5p binds to circ_0009910 and may be involved in the role of circ0009910 in PA progression.

### miR-106b-5p Inhibits Pituitary Adenoma Cell Proliferation, Invasion, Migration, and Epithelial–Mesenchymal Transition In Vitro

The miR-106b-5p mimic and miR-106b-5p inhibitor were transfected into GH3 cells using small interfering RNA (siRNA; Fig. 4C ). EdU and Transwell results showed that miR-106b-5p significantly inhibited GH3 cell proliferation, invasion, and migration ( Fig. 4D , E ).

**Fig. 5:**
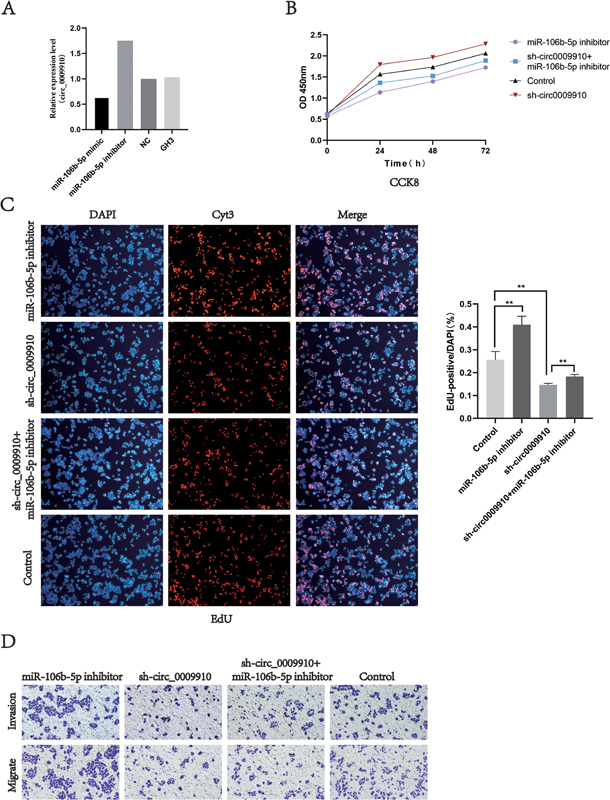
miR-106b-5p partially rescued the function of circ_0009910. **(A)** qRT-PCR detection of miR-106b-5p expression in circ_0009910 after miR-106b-5p transfection of GH3 cells. **(B, C)** Knockdown of miR-106b-5p expression partially reduced the pro-proliferative effect of sh_circ_0009910. **(D)** Knockdown of miR-106b-5p expression partially reduced the pro-proliferative and pro-invasive effects of sh_circ_0009910. ** *p* < 0.01, *** *p* < 0.001. CCK-8, Cell Counting Kit-8; NS, no statistical difference; qRT-PCR, quantitative reverse transcription polymerase chain reaction.

The qRT-PCR results showed that the expression level of circ0009910 in GH3 cells changed with the level of miR-106b-5p ( Fig. 5A ). To verify whether circ_0009910 regulates PA cell progression through miR-106b-5p, we designed rescue experiments. The miR-106b-5p inhibitor was transfected into sh_circ0009910–2 GH3 cells, and the proliferation, invasion, and migration of each group of cells were evaluated. The results showed that the diminished proliferation, invasion, and migration of GH3 cells caused by circ0009910 knockdown could be partially reversed by miR-106b-5p downregulation ( Fig. 5B – D ). The above results suggest that circ0009910 can act as a molecular sponge for miR-106b-5p to play a procarcinogenic role in PA.

### Exosome-Derived circ0009910 Promotes Epithelial–Mesenchymal Transition in GH3 Cells via the miR-106b-3p/STAT3 Axis

Previous studies have shown that STAT3 is a molecule closely associated with tumor epithelial–mesenchymal transition (EMT), 21 and in conjunction with the present study, we further explored whether exosome-derived circ0009910 induces PA cells to undergo EMT through the miR-106b-5p/STAT3 axis.

**Fig. 6:**
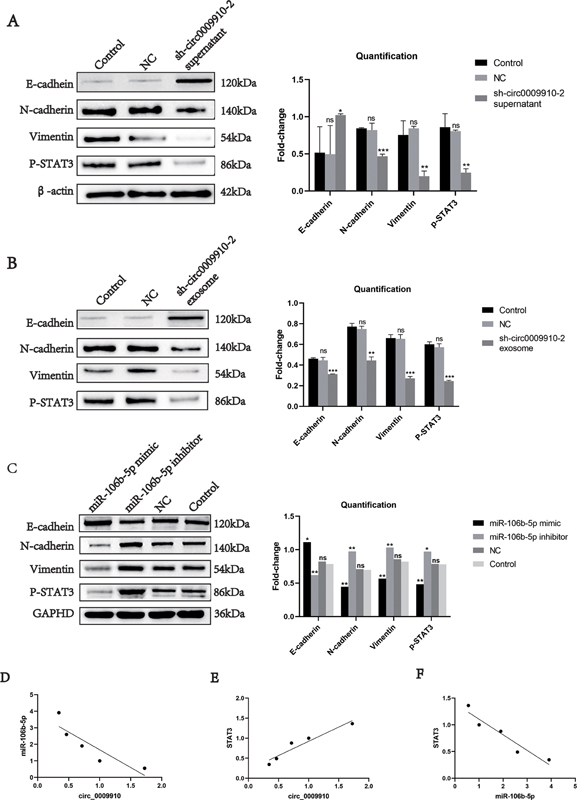
Relationship and correlation analysis of circ0009910, miR-106b-5p with STAT3 and EMT. **(A)** p-STAT3 and N-cadherin were reduced and E-cadherin was upregulated in GH3 cells after sh_circ0009910 intervention. **(B)** EMT-related proteins were expressed at levels consistent with cells in the nude mice hormonal tissues. **(C)** Transfection of miR-106b-5p mimic increased E-cadherin expression and decreased p-STAT3, N-cadherin, and Vimentin expression. **(D–F)** In PA tissues, circ_0009910 was negatively correlated with miR-106b-5p and positively correlated with STAT3, while miR-106b-5p was negatively correlated with STAT3. Statistical analyses of all groups in **(A** – **C)** were compared with the Control group. * *p* < 0.05, ** *p* < 0.01, *** *p* < 0.001. EMT, epithelial–mesenchymal transition; NS, no statistical difference; P-STAT3.

The cells were divided into three groups: GH3 cells treated with sh_circ0009910–2 GH3 cell culture medium, GH3 cells treated with NC group culture medium, and normal GH3 cells. The expression levels of EMT-related proteins and phosphorylated STAT3 were determined by WB in each group. The results showed that p-STAT3 and N-cadherin decreased while E-cadherin increased in the GH3 cells treated with sh_circ0009910–2 GH3 cell culture medium, and the same trend was also shown in the tissues of nude mice with homozygous tumors ( Fig. 6A , B ), whereas inhibition of miR-106b-5p expression partially reversed EMT progression ( Fig. 6C ). Taking the above experimental results together, we can conclude that exosome-derived circ0009910 promotes EMT in PA through the miR-106b-5p/STAT3 axis.

### Correlation Analysis

Pearson's statistical method was used to further explore the correlation of the above molecules in PA tissues. qRT-PCR confirmed that circ0009910 was negatively correlated with miR-106b-5p in PA tissues, circ0009910 was positively correlated with STAT3, and miR-106b-5p was negatively correlated with STAT3 ( Fig. 6D , E, F).

Taken together, the results of this experiment suggest that exosome-derived circ0009910 promotes PA cell proliferation, invasion, and migration and regulates GH secretion levels and the EMT process through the miR-106b-5p/STAT3 axis.

## Discussion

**Fig. 7:**
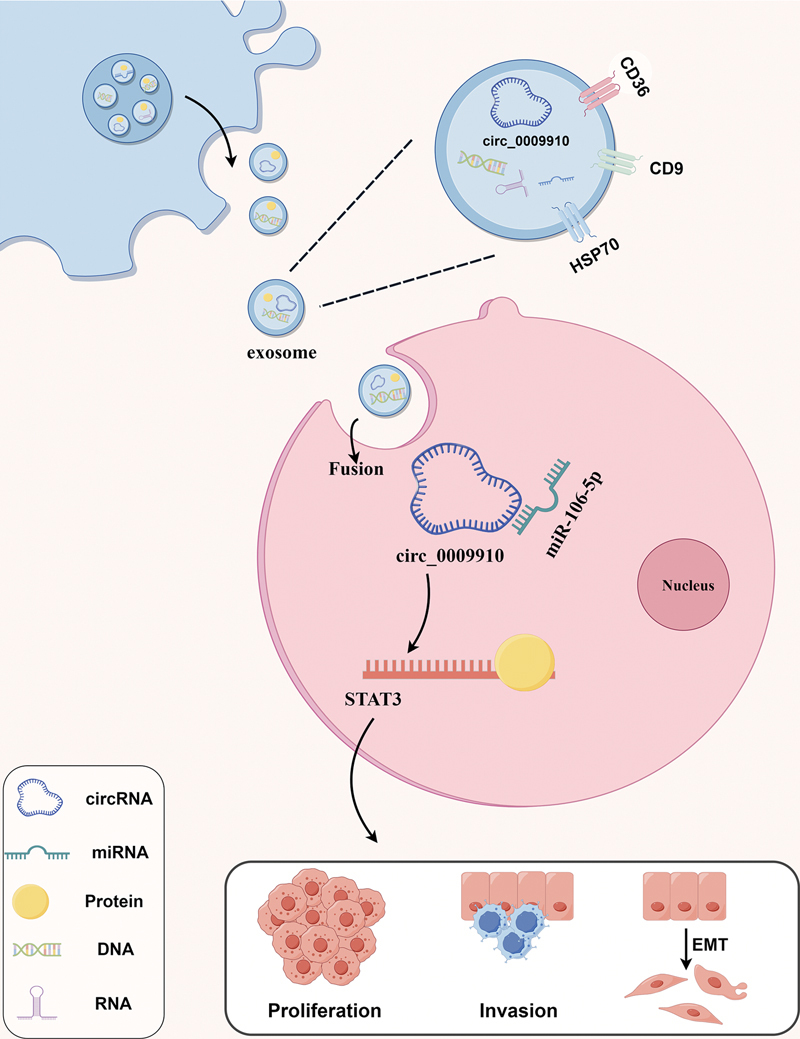
Pattern of exosome-derived circ_0009910 functioning through the miR-106b-5p/STAT3 pathway.

IPAs are a class of sellar tumors with a relatively poor prognosis. The pathogenesis of these tumors is very complex, and the lack of clinically effective molecular markers substantially limits the early diagnosis and treatment of the disease. In this study, we found that the expression level of circ0009910 was elevated in serum exosomes of patients with IPA, and knockdown of circ0009910 could inhibit the proliferation, invasion, and migration of PA cells in vivo and in vitro and regulate the progression of EMT. miR-106b-5p is a molecular sponge for circ0009910, and circ0009910 acts as a molecular sponge for PA by regulating STAT3 to regulate the adverse biological behavior of PA ( Fig. 7 ).

Circular RNAs (circRNAs) are a class of single-stranded noncoding RNAs that can form a unique ring structure by reverse splicing. 22 The lack of a 5′ or 3′ end prevents them from being digested by RNase R. Therefore, circRNAs are more stable and conserved than other noncoding RNAs. 23 With the development of high-throughput sequencing and bioinformatics analysis technologies, an increasing number of studies have shown that the differential expression of circRNAs plays an important role in tumor development and has the potential to serve as a molecular marker. 24 Exosomes are extracellular vesicles rich in genetic material that act as mediators of intercellular communication and can transport their contained circRNAs, miRNAs, and other substances to target cells to play a role in promoting or suppressing tumor. 10 Liu et al. reported that exosomes secreted by hepatocellular carcinoma cells promote distant metastasis through the delivery of circ_MMP2. 25 Yang et al. showed that exosomes derived from bladder cancer cells harbor circTRPS1, which regulates reactive oxygen species homeostasis in cancer cells through activation of GLS3. 26 Ding et al. found that exosomes secreted by drug-resistant glioma cells have higher expression levels of circ_0072083, a process that mediates tumor drug resistance. 27 Exosomes also play an important role in PA. Zhao et al. found that miR-149-5p and miR-99a-3p were overexpressed in exosomes derived from PA cells, a process that inhibits the proliferation and invasion of PA. 28 Zhang et al.'s experimental results showed that lnc H19 derived from exosomes derived from PA cells could shuttle in vivo to inhibit the growth of distal tumor cells. 29

In this study, we illustrate the important protumorigenic role played by exosome-derived circ0009910 in the proliferation, invasion, migration, and EMT of PA both in vitro and in vivo. We performed experiments in rat PA cells, and the results showed that downregulation of exosomal circ0009910 expression in PA cells inhibited normal PA cell proliferation, invasion, and migration, lowered GH levels, inhibited the expression of p-STAT3, N-cadherin, and mesenchymal markers (Vimentin), and promoted the expression of epithelial markers (E-cadherin). Combined with our previous studies, the data identified miR-106b-5p as a possible downstream molecule of circ0009910 by biosignature analysis, and the experimental results showed that the oncogenic effect of circ0009910 could be partially rescued by miR-106b-5p. Therefore, we suggest that circ0009910 is contained in exosomes to accomplish intercellular delivery, and silencing circ0009910 can partially inhibit PA cell proliferation, invasion, migration, and EMT and reduce GH levels via the miR-106b-5p/STAT3 axis.

In this study, we found that the expression level of circ0009910 in serum exosomes was higher in patients with IPA than in patients with noninvasive PA, so we hypothesized that the high expression of exosomal circ0009910 might be related to the invasion of PA. Normal PA cells treated with sh_circ0009910–2 PA cell supernatant showed a significant decrease in invasive and migratory abilities, an increase in the expression of epithelial markers (E-cadherin), and a decrease in the expression of N-cadherin and vimentin. In vitro animal experiments have shown that exosomal circ0009910 knockdown inhibits the tumor growth rate and ultimately reduces tumor size. In hepatocellular carcinoma, the enhanced proliferation, invasion, and migration of cancer cells were associated with the high expression of circ0009910, which directly targets the miR-335-5p/Rho associated with coiled coil binding protein kinase 1 axis to promote the malignant biological behaviors of tumor cells. circ0009910 knockdown resulted in the inhibition of hepatocellular carcinoma cell proliferation, invasion, and migration, 30 which is similar to our experimental results. circRNAs can function in tumors in a variety of ways, and one widely reported function is acting as molecular sponges for miRNAs by adsorbing specific miRNAs and thus preventing them from interacting with target mRNAs, which is known as the competitive endogenous RNA (ceRNA) mechanism. 23 Li et al. reported that in ovarian cancer, circ_0009910 can adsorb miR-145 and play a procancer role through the NF-κB pathway in ovarian cancer. 31 Similarly, Liu et al. reported that circ0009910 can bind to miR-361-3p in gastric cancer and promote proliferation, invasion, and glycolysis of gastric cancer cells through the regulation of small nuclear ribonucleoprotein A. 14 The above results suggest that circ0009910 may also play a role in PA through the ceRNA mechanism.

Therefore, we further explored miRNAs and mRNAs associated with exosome-derived circ0009910 in PA. Our past studies have identified STAT3 as a molecule that is closely associated with PA invasion, and Zhang et al.'s study also demonstrated that activated STAT3 promotes GH PA proliferation and invasion. 32 Zhou et al.'s study in PA showed that STAT3 induced GH transactivation and expression by binding to the GH promoter, and at the same time, overexpressed GH induced intracellular STAT3 phosphorylation and nuclear translocation. The abovementioned mechanisms formed a positive feedback loop that further promoted GH hypersecretion in GH PA. 18 Our results show that silencing circ0009910 reduces the level of GH in cell cultures while downregulating the expression of p-STAT3. This finding suggests that the oncogenicity of circ0009910 may be partially dependent on the STAT3 pathway. Applying bioinformatics analysis techniques to analyze the miRNAs that may exist between circ0009910 and STAT3 and qRT-PCR to verify their binding, we found that miR-106b-5p is the miRNA that binds to circ0009910. Further investigating the effect of miR-106b-5p on PA cells, we found that overexpression of miR-106b-5p can inhibit the proliferation, invasion, and migration of PA cells, decrease the expression of N-cadherin, vimentin, and p-STAT3, and increase the expression of E-cadherin. The results of Wang et al. showed that miR-106b-5p functions in breast cancer by binding to the 3′UTR of CNN1 to promote pulmonary metastasis. 33 Yang et al.'s study in abdominal aortic aneurysm showed similar results to ours, and he found that miR-106b-5p could promote abdominal aortic aneurysm formation by binding to STAT3. 34 Liu et al.'s study found that circ_CCNB1 could act as a molecular sponge for miR-106b-3p by signaling through the AKT/ERK pathway to inhibit GPM1A expression to promote hepatocellular carcinoma progression. 35 Therefore, we suggest that exosome-derived circ0009910 may play a procancer role through binding to STAT3 by acting as a molecular sponge with miR-106b-5p.

In this study, we initially elucidated the important role of exosome-derived circ0009910 in PA, but we were unable to determine whether circ0009910 was the top differentiated molecule in the serum exosomes of patients with IPA versus those with noninvasive PA, and subsequent RNA-seq will provide more accurate conclusions.

Most studies on circRNAs in PA have elucidated the ceRNA mechanisms of different circRNAs. circRNAs are functionally rich, and their study in PA should not be limited to ceRNA mechanisms. A review on circRNAs extensively describes the possible mechanisms by which circRNAs function. In addition to acting as molecular sponges, circRNAs play important roles in maintaining stemness in embryonic and adult stem cells, mediating stem cell differentiation, regulating the cell cycle, regulating apoptosis, mediating autophagy, mediating tumor angiogenesis, and many other aspects. 36 The mechanism of PA is very complex, and the mechanism of circ0009910's role in PA needs further in-depth study.

In summary, this paper shows that circ0009910 expression is upregulated in serum exosomes of patients with IPA. Exosome-mediated circ0009910 promotes PA cell proliferation, invasion, migration, GH secretion, and EMT through the miR-106b-5p/STAT3 axis. Our findings enhance the understanding of the progression of PA invasion, and serum exosomal circ0009910 may be a potential biomarker for patients with IPAs.

## Conclusion

Overall, the present study further explored the molecular mechanisms by which exosome-derived circ0009910 promotes PA progression via miR-106b-5p/STAT3, and these findings underscore the potential of serum exosome circ0009910 as an important biomarker for predicting IPA.
