# Supplementary material for: Exosome-Derived circ0009910 Promotes Pituitary Adenoma Cell Proliferation, Invasion, Migration, and EMT through the miR-106b-5p/STAT3 Axis
Source: J Neurol Surg A Cent Eur Neurosurg. 2025 Sep 18;87(2):99–114. doi: 10.1055/a-2599-4212 (PMC12912908; doi:10.1055/a-2599-4212)
Supplement: Supplementary file 1 — Supplementary Material [file 10-1055-a-2599-4212-s24maroa0067.pdf]

Supplementary Table S1 Sequences of primers used for quantitative RT-PCR assay

Primer ID	Primer sequences (5′–3′)
circ_0009910 F	CCGCGCAATGTCCCTGC
circ_0009910 R	TGCATTCACCTCAGCCATGT
miR-106b-5p F	AAATGCTCATAAAGTGCTGACAGT
miR-106b-5p R	TATGGTTTTGACGACTGTGTGAT
Rat-GAPDH-F	GACATGCCGCCTGGAGAAAC
Rat-GAPDH-R	AGCCCAGGATGCCCTTTAGT
HACTB F	AACCGCGAGAAGATGACCCAG
HACTB R	GGATAGCACAGCCTGGATAGCAA

Supplementary Table S2 Antibody information used in immunohistochemistry and Western blot

Name	Number	Source	IHC	WB	Company
GH	RP1023	Rabbit multiple antibodies	1:200	–	BOSTER
PRL	M00601–3	Mouse monoclonal antibody	1:50	–	BOSTER
Ki-67	A00254	Rabbit multiple antibodies	1:200	–	BOSTER
P53	BM0101	Mouse monoclonal antibody	1:200	–	BOSTER
PIT-1	A03537–1	Rabbit multiple antibodies	1:200	–	BOSTER
ER	MA0057	Mouse monoclonal antibody	1:100	–	BOSTER
GATA-2	BM4342	Rabbit monoclonal antibody	1:20	–	BOSTER
STAT3	PB0540	Rabbit multiple antibodies	1:200	–	BOSTER
p-STAT3	BM4835	Rabbit multiple antibodies	–	1:1,000	BOSTER
E-cadherin	PB9561	Rabbit multiple antibodies	–	1:1,000	BOSTER
N-cadherin	A01577–3	Rabbit multiple antibodies	–	1:1,000	BOSTER
Vimentin	BM0135	Mouse monoclonal antibody	–	1:1,000	BOSTER
GAPDH	BM1623	Mouse monoclonal antibody	–	1:2,000	BOSTER
β-actin	BM0627	Mouse monoclonal antibody	–	1:2,000	BOSTER
Secondary antibodies	BA1054	HRP-goat anti-rabbit	1:5,000	1:5,000	BOSTER
Secondary antibodies	BA1050	HRP-sheep anti-mouse	1:5,000	1:5,000	BOSTER

Abbreviations: GH, growth hormone; IHC, immunohistochemistry; PRL, prolactin; WB, Western blot.

 © 2025. The Author(s).
